# In vitro and numerical simulation of blood removal from cerebrospinal fluid: comparison of lumbar drain to Neurapheresis therapy

**DOI:** 10.1186/s12987-020-00185-5

**Published:** 2020-03-16

**Authors:** Mohammadreza Khani, Lucas R. Sass, M. Keith Sharp, Aaron R. McCabe, Laura M. Zitella Verbick, Shivanand P. Lad, Bryn A. Martin

**Affiliations:** 1 Department of Biological Engineering, The University of Idaho, 875 Perimeter Drive, MS 0904, Moscow, ID 83844-0904 USA; 2 Department of Mechanical Engineering, University of Louisville, 332 Eastern Pkwy, Louisville, KY 40292 USA; 3 Minnetronix Neuro, Inc., 1635 Energy Park Dr, Saint Paul, MN 55108 USA; 4 Department of Neurosurgery, Duke University School of Medicine, 3100 Tower Blvd, Durham, NC 27707 USA

**Keywords:** Computational fluid dynamics, In-vitro model, Subarachnoid hemorrhage, Neurapheresis therapy, Cerebrospinal fluid filtration, Multiphase simulation

## Abstract

**Background:**

Blood removal from cerebrospinal fluid (CSF) in post-subarachnoid hemorrhage patients may reduce the risk of related secondary brain injury. We formulated a computational fluid dynamics (CFD) model to investigate the impact of a dual-lumen catheter-based CSF filtration system, called Neurapheresis™ therapy, on blood removal from CSF compared to lumbar drain.

**Methods:**

A subject-specific multiphase CFD model of CSF system-wide solute transport was constructed based on MRI measurements. The Neurapheresis catheter geometry was added to the model within the spinal subarachnoid space (SAS). Neurapheresis flow aspiration and return rate was 2.0 and 1.8 mL/min, versus 0.2 mL/min drainage for lumbar drain. Blood was modeled as a bulk fluid phase within CSF with a 10% initial tracer concentration and identical viscosity and density as CSF. Subject-specific oscillatory CSF flow was applied at the model inlet. The dura and spinal cord geometry were considered to be stationary. Spatial–temporal tracer concentration was quantified based on time-average steady-streaming velocities throughout the domain under Neurapheresis therapy and lumbar drain. To help verify CFD results, an optically clear in vitro CSF model was constructed with fluorescein used as a blood surrogate. Quantitative comparison of numerical and in vitro results was performed by linear regression of spatial–temporal tracer concentration over 24-h.

**Results:**

After 24-h, tracer concentration was reduced to 4.9% under Neurapheresis therapy compared to 6.5% under lumbar drain. Tracer clearance was most rapid between the catheter aspiration and return ports. Neurapheresis therapy was found to have a greater impact on steady-streaming compared to lumbar drain. Steady-streaming in the cranial SAS was ~ 50× smaller than in the spinal SAS for both cases. CFD results were strongly correlated with the in vitro spatial–temporal tracer concentration under Neurapheresis therapy (R^2^ = 0.89 with + 2.13% and − 1.93% tracer concentration confidence interval).

**Conclusion:**

A subject-specific CFD model of CSF system-wide solute transport was used to investigate the impact of Neurapheresis therapy on tracer removal from CSF compared to lumbar drain over a 24-h period. Neurapheresis therapy was found to substantially increase tracer clearance compared to lumbar drain. The multiphase CFD results were verified by in vitro fluorescein tracer experiments.

## Background

A detailed understanding of cerebrospinal fluid (CSF) physiologic function may help improve treatment of CSF-related central nervous system (CNS) diseases and debilitating neurological conditions. CSF is a clear, colorless fluid that occupies the subarachnoid space (SAS) and the ventricular system within the brain [1]. CSF is believed to be primarily produced within the ventricles of the brain by secretory epithelial cells which form the choroid plexuses and absorbed at the arachnoid granulations located in the SAS on the surface of the superior sagittal sinus [2]. CSF moves with a net flow direction outward from the ventricles to the SAS but also multi-directionally with an oscillatory motion, driven by cardiac and respiratory-related pressure fluctuations and other transient maneuvers [3]. CSF serves multiple physiological functions that continue to be discovered. Some of the roles of CSF include: (1) suspension of the delicate brain tissue by the Archimedes principle making the brain tissue nearly neutrally buoyant, (2) damping of forces that act on the brain tissue due to transient impact [4], (3) providing immunological and biochemical homeostasis for the CNS [5], and (4) delivery of metabolites and micronutrients to the CNS [1, 6].

The importance of CSF dynamics has been investigated in several CNS diseases that include neuroinflammatory conditions such as multiple sclerosis [7, 8] and neurovascular conditions such as cerebral ischemia [9, 10], traumatic brain injury [11] and subarachnoid hemorrhage (SAH) [12]. Delayed ischemia and hydrocephalus following SAH are two of the primary causes of morbidity/mortality due to the presence of blood in the subarachnoid spaces (SAS) [13–15]. Therefore, strategies to facilitate the rapid clearance of blood from the SAS may reduce the risk of these complications to patients. Such strategies, including lumbar drain [12, 16], cisternal drainage [17, 18] and cisternal lavages [19, 20], have been studied by a number of investigators, but the exact biological mechanisms responsible for delayed ischemia are not yet clear.

Neurapheresis™ therapy (Minnetronix Neuro, Inc., St. Paul, MN) is being investigated as a means to potentially rapidly remove red blood cells from the SAS and consequently decrease the incidence or severity of secondary complications. In brief, Neurapheresis therapy involves aspiration of CSF from the lumbar spinal SAS, filtration of CSF and removal of red blood cells and detritus to a waste bag, and then return of filtered CSF to the SAS at the thoracic spine. The different locations of the aspiration and return ports may facilitate advective bulk movement of CSF between the ports within the SAS. Additional details on Neurapheresis therapy are provided by Khani et al. [21].

Computational and in vitro modeling constitute potential methods to study complex transport phenomena occurring during blood clearance from CSF spaces and, subsequently, to improve devices and protocols to treat neural disorders. Clinical trials to develop guidelines for more effective clearance are difficult due to limited availability of subjects and associated study costs. Empirical models limit our ability to investigate CSF filtration technologies, highlighting the need for a computational tool. Human clinical trials of Neurapheresis therapy in SAH patients are being conducted (PILLAR trial [22–24]), but these studies lack real-time visualization of blood distribution and are only able to sample CSF from select locations. Real-time visualization is important in this study because it helps us to monitor the tracer concentration at each location and time frame and then use it to predict the total the time needed to completely remove all the blood from CSF system for a subject-specific patient. Results from this real-time visualization could be used to estimate and compare the efficiency of the Neurapheresis therapy to traditional lumbar drain at each time point. Also, in principle, a nonhuman primate experimental model of SAH could be developed, but such studies are expensive, are only available at limited research centers, and do not provide similar CSF dynamics to humans [25, 26].

Several in vivo, in vitro, and in silico studies have been conducted to better understand Neurapheresis therapy. A rabbit model was used to investigate Neurapheresis therapy in the context of cryptococcal meningitis. This study showed a 5-log reduction in yeast concentration and 1-log reduction in its polysaccharide antigen over 24-h. A drawback of the study was that the rabbit model has an extremely small SAS, thus it is unclear how rabbit CSF dynamics compare to humans [27]. A study by Tangen et al. [28] presented a computational and in vitro model of SAH clearance from CSF. This study provided information about the potential of CSF filtration to assist with blood removal under different body orientations (i.e., supine versus upright) with an idealized representation of CSF space anatomy. Khani et al. [21] formulated a numerical model to investigate the impact of Neurapheresis therapy on CSF flow velocities in a realistic spinal SAS geometry with subject-specific CSF flow along the spine. However, this model did not take into account the intracranial portion of the CSF system and lacked a multiphase fluid mixture of blood and CSF.

While the previous studies provided insight into Neurapheresis therapy, they had the following important limitations: (a) they lacked realistic system-wide CSF geometry, (b) they did not consider the multi-phase solute transport within the CSF, (c) they were conducted over relatively short time periods, (d) they utilized animal models that have different CSF dynamics than humans, and/or (e) they lacked numerical model solution verification. This study seeks to address these limitations by formulating a multi-phase numerical model of CSF system-wide solute transport within an anatomically realistic human geometry and by verifying the numerical solution versus in vitro measurements.

## Methods

The overall approach was to build a subject-specific multiphase computational fluid dynamics (CFD) model and to verify model results using a corresponding bench-top model. After verification, the models were used to quantitatively assess tracer removal by Neurapheresis therapy compared to lumbar drain. Fluorescein solution was used as a surrogate tracer and modeled as a bulk fluid phase within the CSF [29–32] to track clearance of blood components, specifically hemoglobin derivatives.

### Model geometry

**Fig. 1:**
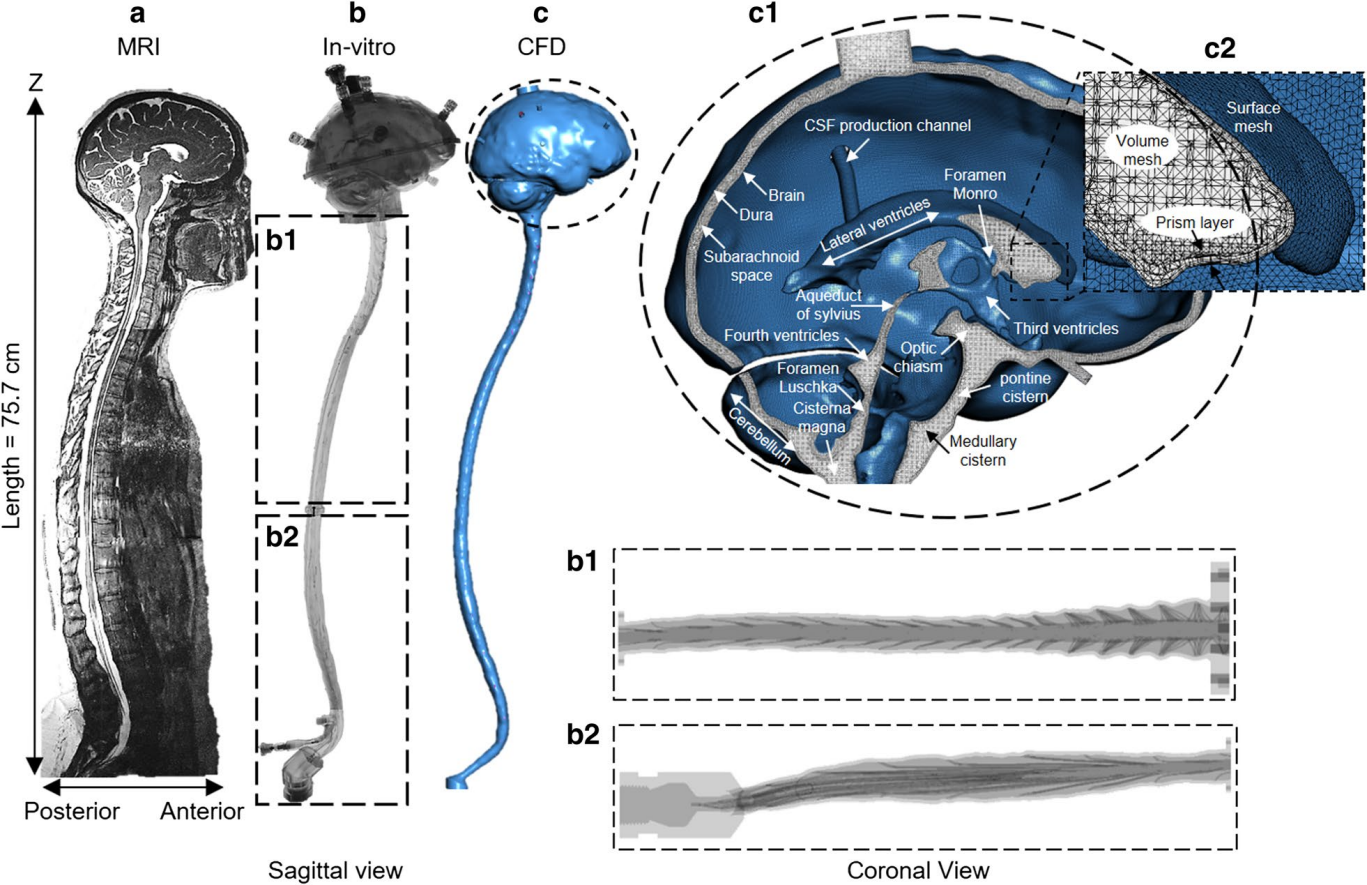
Overview of in vitro and numerical model based on subject specific MRI measurements. **a** T2-weighted MR image of the entire CSF space for the human analyzed to acquire subject-specific anatomy and natural CSF pulsations. **b** An in vitro bench-top model of the human CSF filled spaces was generated from MRI images, (b1–2) in vitro model spinal canal including nerve roots and cauda equina. **c** computational model of the human central nervous system (c1) magnification of the cranial SAS consisting of lateral ventricles, foramen Monro (left and right), third ventricle, aqueduct of Sylvius, 4th ventricle, foramen Luschka, cisterna magna, pre-pontine and pontine cistern, trigeminal cistern, quadrigeminal cistern, Sylvian cisterns (left and right), and cortical subarachnoid space. (c2) Volumetric and surface mesh visualization with prism layers near the wall

A high-resolution T2-weighted magnetic resonance imaging (MRI) sequence was used to acquire a subject-specific geometry of the CSF system of a 23-year-old healthy female subject (Fig. 1a). This model contained two main regions: (1) the spinal SAS and (2) the intracranial CSF space. The MRI protocol and image reconstruction method for the spinal SAS were previously described by Sass et al. [33]. In brief, the spine model combined the high-resolution MR imaging with anatomic measurements and cadaveric studies in the literature and included 31 pairs of anatomically realistic spinal cord nerve roots, the filum terminale and the thecal sac (Fig. 1b).

A detailed intracranial CSF space geometry obtained with high-resolution MRI was added to the spinal SAS. The CSF space schematic in this region is shown in Fig. 1c. The intracranial CSF was manually segmented using ITK-SNAP (Version 3.4.0, University of Pennsylvania, USA), exported in .STL (stereolithography) format, and imported into Blender (Amsterdam, Netherlands, Version 2.79). The model geometry was simplified within Blender to include the following key intracranial CSF spaces: (a) lateral ventricles and foramen of Monro (left and right), (b) third ventricle and aqueduct of Sylvius, (c) fourth ventricle and foramen of Luschka (lateral apertures), (d) cisterna magna, (e) pre-pontine and pontine cisterns, (f) trigeminal cistern, (g) quadrigeminal cistern, (h) Sylvian cisterns (left and right), and (i) cortical SAS (Fig. 1c1). The complex geometry of the cortical surface and the ventricular CSF system was smoothed and simplified such that the total cranial CSF volume remained equivalent to that of the original segmentation. The cortical SAS was divided at the midline to represent the falx cerebri and superior sagittal sinus. The posterior-superior aspect of the cerebellar cistern was terminated by a barrier representing the tentorium cerebelli. The cortical SAS was considered to cover the entire cortical surface with a uniform thickness of ~ 2.78 mm. To represent CSF production from the choroid plexus, CSF production ports with diameters of 5 mm were added to the superior aspect of the lateral ventricles (Fig. 1c1). A 5.5 mm diameter port was also added at the lumbar spine at the L3–L4 for catheter insertion. Each partitioned cistern was exported as a stereolithography (.STL) file and imported into Autodesk Netfabb (San Rafael, CA, Build 1608) to ascertain volume, surface area, and connectivity. The superior and inferior aspects of the model incorporated 22.5 mm diameter cylindrical passages with a length of 8 mm to accommodate oscillatory CSF pulsation to the system.

**Fig. 2:**
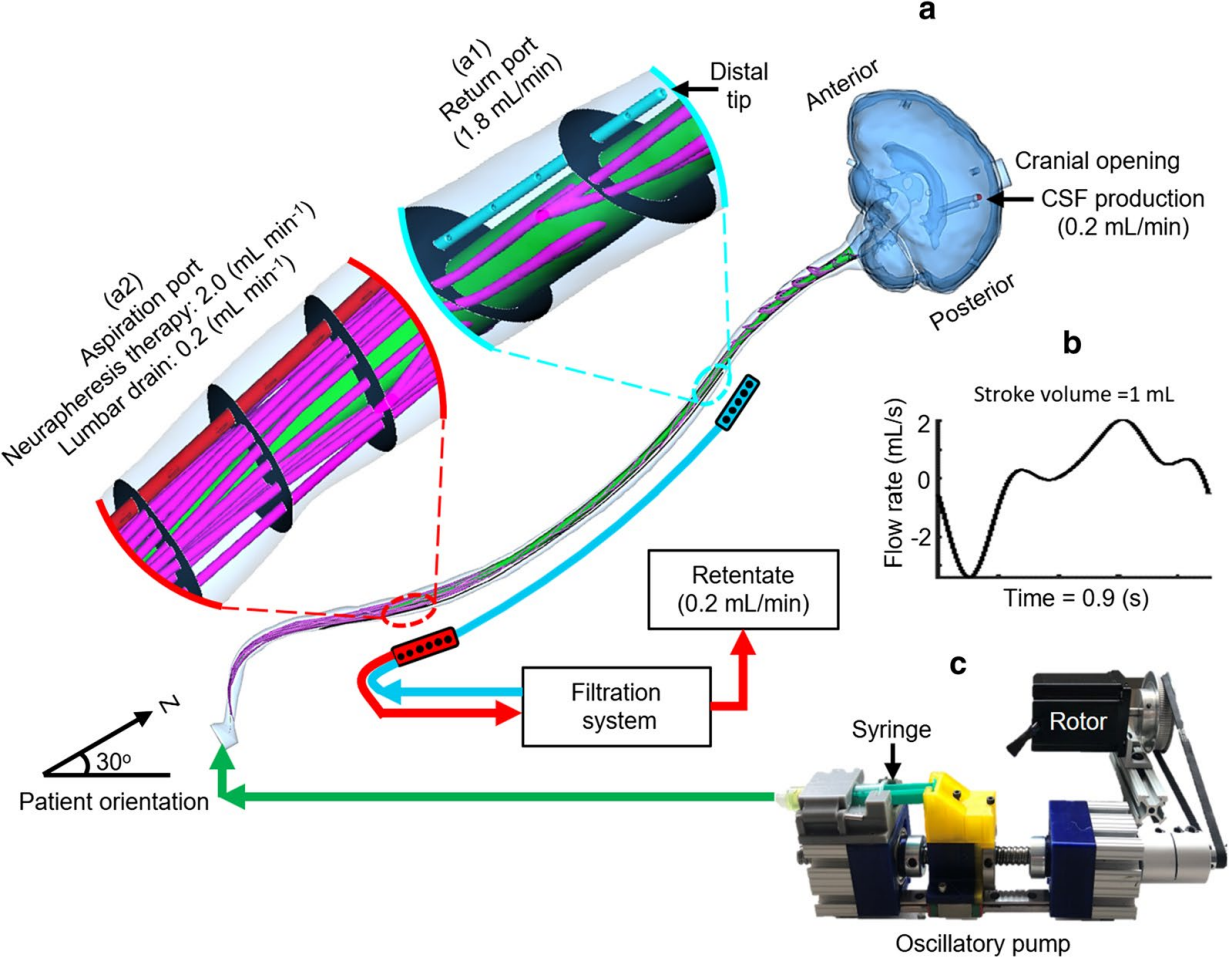
Schematic of the Neurapheresis system and study protocol. **a** Three-dimensional CFD model of the SAS with flow boundary conditions and magnified view of the Neurapheresis catheter return and aspiration ports. **b** Oscillatory pump to induce CSF pulsations to match the CSF flow field acquired by phase contrast-MRI

The dual-lumen Neurapheresis catheter geometry was identical with our previous publication [21]. In brief, the catheter was inserted at L3–L4 and located within the posterior SAS with the aspiration port at L2 and the return port at T2 vertebral level (Fig. 2a). Internal and external catheter diameters were 0.5 and 0.7 mm, respectively, for the inner lumen, and 1.7 and 1.5 mm for the outer lumen. A series of 11 holes were located at the return port and 12 holes at the aspiration port (Fig. 2a1, a2).

### Computational fluid dynamic model

#### Computational mesh

An unstructured tetrahedral computational mesh was generated using ANSYS ICEM 19.2 (Canonsburg, PA). Details on the prism layer and Mesh parameters are included in Table 1 and Fig. 1c2. The mesh was refined near the catheter aspiration and return ports, since at these regions the geometric dimensions of the holes were relatively small. The mesh included the internal geometry of the return and aspiration ports to ensure correct flow distribution from each hole. The final mesh covering the Neurapheresis catheter, cranial, and spinal SAS had 16.8 million cells. To allow verification of numerical results, an identical geometry was used for the in vitro model (Fig. 1b).

**Table 1:** Mesh details

Parameter	Type
Cell type	Tetrahedral
Face type	Triangle
Prism layer	4
Prism height	0.05 (mm)
Growth law	Exponential
Growth factor	1.2
Mesh cells	16.8 M
Mesh faces	26.4 M
Mesh nodes	3.7 M
Min mesh size	0.05 (mm)
Max mesh size	1.0 (mm)

Time step: 0.1 (s), cycle: 5th, time: 100 (s)

#### Boundary conditions

Clearance of tracer after SAH with Neurapheresis therapy and lumbar drain was studied under the following boundary conditions (Fig. 2a). The model system was oriented at 30° to horizontal to mimic a typical patient position within a hospital bed. Fluid was aspirated and returned under Neurapheresis therapy at 2.0 and 1.8 mL/min. The 0.2 mL/min difference between return and aspiration matched the drain rate applied under lumbar drain (see details below). Constant CSF production from the choroid plexus within the left and right ventricles was specified to be 0.1 mL/min (0.2 mL/min total) at the CSF production channel entrance. For the lumbar drain simulation, a drainage rate of 0.2 mL/min was specified, as this is a nominal value typically observed in the literature for that procedure [34, 35].

To represent CSF pulsation around the brain and spinal cord, an oscillatory velocity inlet boundary condition was defined at the model caudal opening using a User Defined Function. The exact waveform was derived from the C2–C3 CSF flow rate waveform (Fig. 2b) obtained from phase-contrast MR imaging of the healthy 23-year-old female subject [36]. The angular frequency of the waveform was ω = 6.98 s^−1^. A zero-pressure outlet boundary condition was defined at the cranial opening. No-slip boundary conditions were imposed at the model walls (dural, pial and intraventricular spaces) with the walls modeled as stationary. CSF was modeled as incompressible with a density of 998.3 kg/m^3^ and viscosity of 0.89 mpa s. Tracer density and viscosity was assumed to be identical to CSF.

#### Flow model

The pulsatile CSF flow field was computed using ANSYS Fluent 19.2 (Canonsburg, PA) by solving the continuity and Navier–Stokes equations

1 $$\overset{\lower0.5em\hbox{$\smash{\scriptscriptstyle\rightharpoonup}$}} {\nabla } \cdot \left[ {\rho {\mathbf{\overset{\lower0.5em\hbox{$\smash{\scriptscriptstyle\rightharpoonup}$}} {u} }}\left( {{\mathbf{\overset{\lower0.5em\hbox{$\smash{\scriptscriptstyle\rightharpoonup}$}} {x} }},t} \right)} \right] = 0$$

2 $$\rho \frac{{\partial {\mathbf{\overset{\lower0.5em\hbox{$\smash{\scriptscriptstyle\rightharpoonup}$}} {u} }}}}{\partial t} + \rho {\mathbf{\overset{\lower0.5em\hbox{$\smash{\scriptscriptstyle\rightharpoonup}$}} {u} }} \cdot \overset{\lower0.5em\hbox{$\smash{\scriptscriptstyle\rightharpoonup}$}} {\nabla } {\mathbf{\overset{\lower0.5em\hbox{$\smash{\scriptscriptstyle\rightharpoonup}$}} {u} }} = - \overset{\lower0.5em\hbox{$\smash{\scriptscriptstyle\rightharpoonup}$}} {\nabla } p + \overset{\lower0.5em\hbox{$\smash{\scriptscriptstyle\rightharpoonup}$}} {\nabla } \cdot \mu \overset{\lower0.5em\hbox{$\smash{\scriptscriptstyle\rightharpoonup}$}} {\nabla } {\mathbf{\overset{\lower0.5em\hbox{$\smash{\scriptscriptstyle\rightharpoonup}$}} {u} }} + \rho {\mathbf{\overset{\lower0.5em\hbox{$\smash{\scriptscriptstyle\rightharpoonup}$}} {g} }}$$

where $$\rho$$ is fluid density, $$\mu$$ is fluid viscosity, $${\mathbf{\overset{\lower0.5em\hbox{$\smash{\scriptscriptstyle\rightharpoonup}$}} {g} }}$$ is acceleration due to gravity, $${\mathbf{\overset{\lower0.5em\hbox{$\smash{\scriptscriptstyle\rightharpoonup}$}} {u} }}$$ is the velocity vector, and $$p$$ is the pressure field.

#### Multiphase model

The 3D CFD computations used the ANSYS multi-phase fluid model to track the dispersion of a tracer within the CSF, with tracer volume fraction given by

3 $$\frac{\partial }{\partial t}\left( {\alpha_{k} \rho_{k} } \right) + \nabla \cdot \left( {\alpha_{k} \rho_{k} \vec{\upsilon }_{m} } \right) = - \nabla \cdot \left( {\alpha_{k} \rho_{k} \vec{\upsilon }_{dr \cdot k} } \right)$$

where $$q$$ is the bulk fluid phase, $$\rho_{k}$$ and $$\alpha_{k}$$ are the phase density and volume fraction of phase $$k$$, $$\vec{\upsilon }_{m} = \frac{{\sum\nolimits_{k = 1}^{n} {\alpha_{k} \rho_{k} \overset{\lower0.5em\hbox{$\smash{\scriptscriptstyle\rightharpoonup}$}} {u}_{k} } }}{{\rho_{m} }}$$ is the mass-averaged velocity, $$\rho_{m}$$ is the mixture density, and $$\vec{\upsilon }_{{{\text{dr}},k}}$$ is the drift velocity for phase $$k$$, with phase k = 1 being the CSF and k = 2 for the tracer. We assumed the relative velocity (slip velocity) between phase *k* and the bulk fluid to be zero. Thus, the drift velocity was considered to be zero.

Based on clinical SAH observations and previous research, we assumed a baseline tracer concentration of 10% ($\alpha_{2}$ = 0.1) throughout the model domain. Tangen et al. [37] showed that blood debris is evenly dispersed throughout the spinal SAS within the first hour post-SAH. For the present study, we expected at least 1-h of time to have passed before a lumbar drain or Neurapheresis therapy system could be applied post-SAH. The lumbar puncture is a common clinical emergency procedure used to aid in SAH diagnosis [38]. Medical doctors have observed the CSF samples from these SAH patients to be colored with a light pink shade (xanthochromia) [39], indicating that blood has spread throughout the CSF system down to the lumbar spine, justifying the evenly-mixed tracer concentration used in this model as a reasonable approximation. Steady-streaming velocities were determined based on the average of 10 CSF flow cycles with transient effects eliminated by removal of the first flow cycle. Similar to Kuttler et al. [40], the fixed velocity field (“frozen flow field”) was used to solve the volume fraction equation for spatial–temporal tracer concentration. The axial distribution of tracer concentration, $\alpha (z)$, for 3 mm thick slices along the z-axis was computed by:

4 $$\alpha (z) = \frac{{\sum\nolimits_{slice} {\left| {\alpha_{k} (z)} \right|V(z)} }}{{\sum\nolimits_{slice} {V(z)} }}$$

where $V$ is the cell volume and summations computed for all cells within each 3 mm thick cross-section. Spatial–temporal distribution of tracer concentration was plotted over 24-h for Neurapheresis therapy and lumbar drain.

To determine concentration profiles over 24-h after SAH, we determined the solute transport due to the steady-streaming CSF velocity field. Molecular diffusion of large molecules within the CSF (MW ~ 150 kDa) is much smaller than steady-streaming and oscillatory CSF velocities [40]. However, shear and mixing across the cross section has the potential to greatly increase the effective diffusivity in the spinal SAS [41]. Tangen et al. [37] found that molecular diffusion had a negligible impact on tracer spread within an idealized geometry representing the spinal and cranial SAS. Kurtcuoglu et al. [42] also neglected diffusion in their model. To make the computational effort reasonable, molecular diffusion of the tracer was not included in the current study. However, as described in the following paragraphs, the potential impact of neglecting diffusion was estimated.

To help understand the relative importance of diffusive versus advective mass transport, the Sherwood number ($Sh$) was calculated and $Sh = \frac{h}{{{\mathbf{D}}/L}}$ provides the ratio of convective mass transport, $h\,$, to the effective diffusive mass transport, ${\mathbf{D}}/L$, where $L\,$ is a characteristic length and ${\mathbf{D}}$ is the effective diffusivity including shear-augmented dispersion. $h\,$ was computed based on the mean cross-sectional velocity at peak systolic (h = 0.26 m/s and 2.4 m/s for cortical and spinal SAS, respectively). $L\,$ was assumed to be the minimum gap width between the shells in the cortical SAS (~ 2 mm) and mean of the hydraulic diameter for the spinal SAS (5.87 mm).

To see if the tracer is a good similitude for hemoglobin clearance, ${\mathbf{D}}$ was calculated for both tracer and hemoglobin using an order-of-magnitude model by Sharp et al. [41]:

5 $${\mathbf{D}} = \left( {1 + R_{max} } \right)D$$

6 $$R_{max} = P^{2} Sc/\alpha^{2}$$

7 $$\alpha^{2} = \beta^{2} /Sc$$

8 $$\beta^{2} = (L/2)^{2} \omega /D$$

where $R_{max}$ is the maximum enhancements with optimal mixing,$P$ is the characteristic non-dimensional pressure gradient ($P$ ~ 152.6 [41]), $\alpha$ is Womersley number and $\beta$ is oscillatory Peclet numbers. Schmidt number ($Sc\, = \frac{\nu }{D}$) was described as the ratio of momentum diffusivity of water at room temperature ($\nu$ = 0.89 E−06 m^2^/s), to molecular diffusion coefficient ($D$). The molecular diffusivity of the fluorescein tracer is $D$ = 4.25 E−10 m^2^/s [43, 44] and hemoglobin is $D$ = 10.2 E−11 m^2^/s [45].

#### Solver settings

Simulations were carried out using the PISO Scheme (pressure-implicit with splitting of operators) to solve the flow equations with second-order upwind for momentum discretization, PRESTO! (PREssure STaggering Options) for pressure discretization, and first-order upwind for volume fraction discretization. Default values were used for under relaxation factors. The implicit formulation was used for volume fraction parameters and a dispersed model was used for phase interface modeling. The convergence criteria for velocity, continuity, momentum, and phase volume fraction were set to 1E−06 with time step = 0.1 s and maximum of 100 iterations per time-step. Total simulation time was 14 days to compute 24-h of real-world time in parallel mode with 126 GB RAM and 38 processors. Time required to solve the fixed flow field (11 flow cycles) was 36 h.

#### Numerical sensitivity studies

Axial distribution of average tracer concentration at 1 h was verified by numerical sensitivity studies for time-step size and mesh resolution. Results were computed for a “coarse”, “medium”, and “fine” mesh with wall prism layers. For the medium mesh, time-step sensitivity was then checked with time-step resolution given by fractions of the cardiac cycle, T = 0.9 (s), for T/18, T/9, T/5. Based on these results, a “Medium” mesh with time step = T/9 was carried forward for computation of final results.

### In vitro experimental model

To help verify the numerical model results, an in vitro CSF model was constructed with a fluid domain geometry identical to the numerical model. For the in vitro model, the fluid domain was encased in a 2 mm thick transparent shell. The shell was divided into cranial (Fig. 1b), upper thoracic (Fig. 1b1), and lower spine (Fig. 1b2) pieces not exceeding the maximum build size of the 3D printer. The final digital geometry was submitted for STL 3D printing with a layer and in-plane resolution of 127 μm and 500 μm, respectively, for the cranial volume and 127 μm and 250 μm in the spinal column. The caudal end of the model was connected to a custom-built oscillatory flow pump imparting an identical CSF waveform utilized for the numerical simulation (Fig. 2b). The dual-lumen Neurapheresis catheter was inserted through an access port such that aspiration and return flow occurred at L2 and T2, respectively (Fig. 2a). These locations were congruent with the numerical model.

To mimic SAH blood distribution within the CSF, an aqueous solution of fluorescein sodium (25 mg/1000 mL) was used to fill the entire model representing a uniform distribution of blood. A uniform distribution was assumed because previous research from Tangen et al. [37] showed that blood debris is evenly dispersed throughout the spinal SAS within the first hour post-SAH. Usage of fluorescein as a surrogate tracer for blood is in agreement with previous studies that sought to track blood components in SAH, specifically hemoglobin derivatives [29–32]. Experiments were conducted with deionized water, since the transport of SAH products is thought to be passive, i.e., the effects of chemical reactions are negligible [37]. Fluorescein also does not mimic hydrodynamic effects on finite-size particles, such as the lift exerted on red cells near a boundary.

A continuous flow syringe pump (KD Scientific Legato 270, Holliston, MA) was used to aspirate fluid via the Neurapheresis catheter at 2.0 mL/min. This same pump was also configured in parallel to return fresh deionized water at a rate of 2.0 mL/min. This clean return flow was split and 0.2 mL/min was diverted by a second continuous flow syringe pump for CSF production originating from the ventricles. Thus a net flow of 1.8 mL/min was delivered to the return port of the Neurapheresis catheter. Another syringe pump was used to generate the subject-specific CSF flow wave form (Fig. 2c).The caudal end of the model was chosen as the input location for SAS CSF oscillations. This location allowed a uniform CSF flow rate at any cross-section throughout the rigid subarachnoid space. Also, this location allowed CSF outflow to occur at the top of the brain, a region approximately located near the arachnoid granulations, where CSF outflow is believed to occur into the superior sagittal sinus in vivo. Pump flow rates were verified by a graduated cylinder and stopwatch before each experiment. Identical to the numerical model, all experiments were conducted with the model at 30 degrees (head up) from horizontal.

The following digital image subtraction technique was applied to quantify spatial–temporal tracer concentration. The entire system was enclosed to block ambient light sources. The model was surrounded within the enclosure by blue wavelength (450 (nm)) light emitting diodes triggered to a camera flash. For model calibration, a baseline image was obtained without any tracer introduced to the model. To establish baseline decay of the fluorescein tracer, time-lapse imaging was obtained at 5-min intervals over a total of 24-h with the tracer solution at a known concentration 15 μM. To eliminate aliasing artifacts due to CSF oscillations, images were collected with a trigger set to the cardiac systole. An identical series of images was then collected for the same experiment under Neurapheresis therapy and also with lumbar drain.

Spatial–temporal distribution of tracer concentration for each case was computed as $\alpha = \frac{{I_{\exp } - I_{0} }}{{I_{b} - I_{0} }}$, where $I_{\exp }$ is the signal intensity for each case, $I_{0}$ is the background image intensity at zero concentration and $I_{b}$ is the baseline decay. To account for lens distortion, the distance and orientation of the camera to the model was measured and used to correct axial pixel position for comparison to CFD results. Similar to the CFD simulations, spatial distribution of tracer concentration was averaged for 3 mm thick slices along the model (z-direction).

12-bit images were collected with 12 MP resolution using a digital camera (Sony alpha a7s ii) with a 50 mm focal length prime lens with f/10 aperture (Sony E 50 mm f/1.8 OSS). Imaging settings were 2000 ISO, white balance 5500 K, and 1/100 s shutter speed. A 525 nm bandpass filter (Midwest optical systems BP525-49) was used in front of the lens to improve image signal-to-noise ratio.

### Geometric and hydrodynamic quantification

Based on the 3D reconstruction and meshing, the following geometric and hydrodynamic parameters were calculated along the spine at 1 mm intervals using our previously described methods [46]. Reynolds number based on hydraulic diameter was calculated as $Re = \frac{{\left| {Q_{\text{max} } } \right|D_{H} }}{{\nu A_{cs} }}$, where $\left| {Q_{\text{max} } } \right|$ is the absolute value of the peak flow rate from the flow rate waveform at each cross section, *D*_*H*_ is hydraulic diameter, *A*_*cs*_ is the cross-sectional area and $\nu$ is kinematic viscosity. Womersley number was quantified as $\alpha = \frac{{D_{H} }}{2}\sqrt {\omega /\nu }$ where $\omega = 2\pi /T$ is angular velocity and T is the cycle period. Mean cross sectional velocity at peak systolic and diastolic flow, was computed as $\frac{{Q_{peak} }}{{A_{cs} }}$, with *Q*_*peak*_ defined as the maximum flow rate at peak systole and diastole at each slice.

### Quantification of steady-streaming CSF flow

To quantify steady-streaming, the cyclic mean velocity in the z-direction, $U_{z{\text{-}}mean}$, was computed for each node in the computational mesh as a summation of z-velocity on each node during one cycle similar to our previous publication [25]. $U_{z{\text{-}}mean}$ was visualized at a mid-sagittal slice for Neurapheresis therapy and lumbar drain. A positive value for $U_{z{\text{-}}mean}$ indicates steady-streaming in the rostral direction. $U_{z{\text{-}}mean}$ was used in $Sh$ number calculations as a convective mass transfer term, $h$, since the dominant velocity field in the simulation was assumed to be equal to $U_{z{\text{-}}mean}$. The axial distribution of steady-streaming, $U_{ss} (z)$, was estimated by computing the cross-sectional average of $U_{z{\text{-}}mean}$ magnitude:

9 $$U_{ss} (z) = \frac{{\sum\nolimits_{cell} {\left| {U_{z{\text{-}}mean} (z)} \right|V(z)} }}{{\sum\nolimits_{cell} {V(z)} }}$$

where the summations are over all cells in the cross section at a given z location. $U_{ss} (z)$ was calculated for z-slices at 1 mm intervals along the spine. To further quantify the magnitude of steady-streaming flow, a non-dimensional fraction of the specified flow rate amplitude was defined as:

10 $$Q_{ss(z)} = \frac{{ \, U_{ss} (z)A_{cs} }}{{2Q_{peak} }}$$

### Verification of numerical results

A detailed comparison of numerical and in vitro results was performed by the following correlation analysis similar to that previously conducted by our group [47]. In vitro experiments were conducted using fluorescein as a tracer (as described above) under Neurapheresis therapy and lumbar drain. In vitro and CFD spatial–temporal tracer concentration results were compared by linear regression and Bland–Altman plot analysis with 95% confidence interval calculations.

## Results

Overall, numerical and in vitro results showed agreement in terms of spatial–temporal concentration of tracer over the 24-h simulation period. Neurapheresis therapy was found to clear tracer from the CSF more rapidly than lumbar drain with most of the clearance occurring within the thoracic SAS after 1-h of treatment.

### Geometric and hydrodynamic parameters

**Fig. 3:**
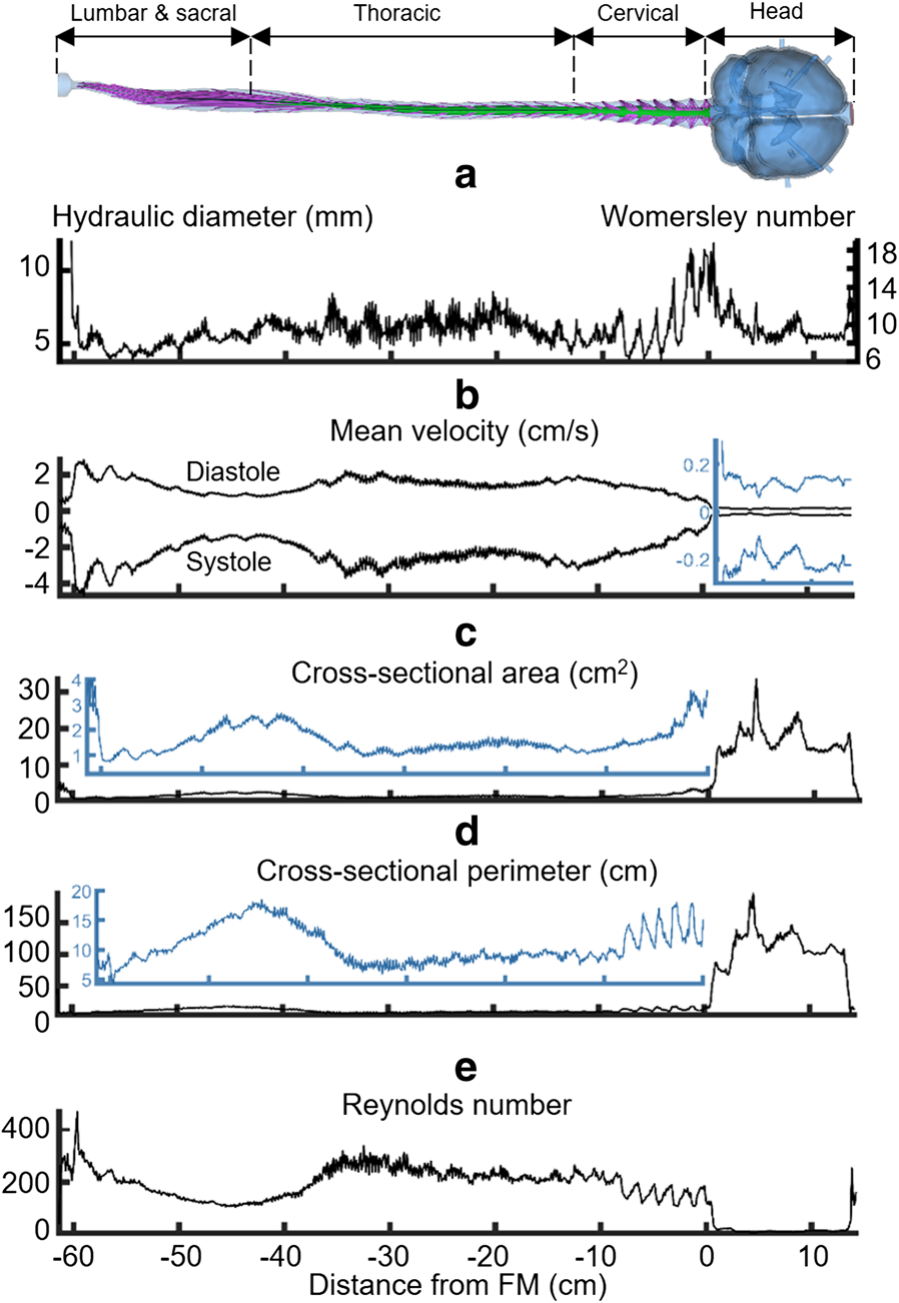
Hydrodynamic and geometric characterization of the computational domain in relation to distance from the foramen magnum (FM). **a** Hydraulic diameter (Left axis) and Womersley number (right axis), **b** mean velocity of CSF, **c** cross-sectional area, **d** cross-sectional perimeter of the subarachnoid space, and **e** Reynolds number

A summary of volumetric parameters for the model is included in Table 2. Total length of the SAS (cranial and spinal) was 75.6 cm. Spinal SAS volume was 100.3 mL and intracranial CSF volume was 221.6 mL. A review of more recent literature using non-invasive MRI-based methods indicates that total CSF volume in healthy adults to range from ~ 250 to 400 cm^3^ [48–52]. The hydraulic diameter and Womersley numbers had an average value of 6.2 mm and 9.8 within SAS (Fig. 3a). Local maxima for hydraulic diameter and Womersley number were located at the foramen magnum. Mean CSF velocity had the greatest values in the lumbar spine at − 4.7 and 2.8 cm/s for the peak systole and diastole, respectively. Minimum of the mean velocity occurred in the cranial SAS (Fig. 3b). Mean cross-sectional perimeter and area were 28.3 cm and 4.2 cm^2^, respectively. As expected, maximum area and perimeter was located at the cranium (Fig. 3c, d). A notable increase in area and perimeter was present at ~ 5 cm cranial to the foramen magnum where the lateral ventricles are located. Maximum Reynolds number was 461 and was located at the caudal end of lumbar spine where the in vitro model tubing entered the system (required for the in vitro model flow pump connection) (Fig. 3e).

**Table 2:** CSF space geometric parameters

Parameter	Volume (mL)
Spinal cord	19.6
Nerve roots	6.0
Dura	125.9
Total spinal CSF	100.3
Cortical SAS	153.6
Ventricular system	19.7
Cerebellar SAS	21.8
Basal cisterns	26.5
Total intracranial CSF	221.6
Total CSF	321.9

Total spinal CSF = dura − (nerve roots + spinal cord)

Total intracranial CSF = cortical SAS + ventricular system + cerebellar SAS + basal cisterns

### Numerical quantification of steady-streaming CSF velocities and dimensionless parameters

**Fig. 4:**
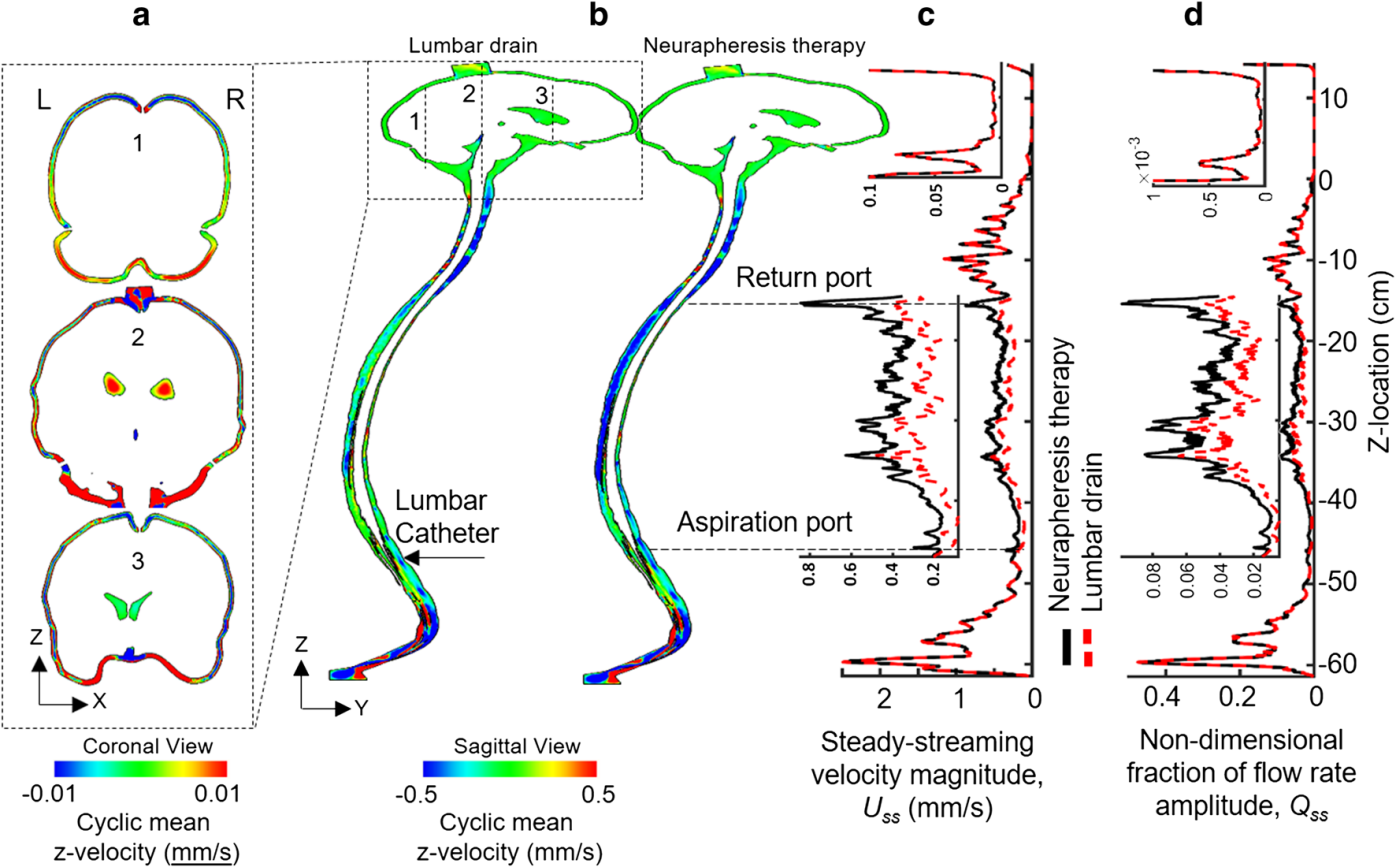
Quantification of steady-streaming velocities. Steady-streaming within the spinal SAS increases with Neurapheresis therapy compared to lumbar drain. **a** Coronal view of cyclic mean z-velocity profiles, $U_{z{\text{-}}mean}$, in the cranial SAS is nearly identical for Neurapheresis therapy and lumbar drain. **b** Sagittal view of cyclic mean z-velocity profiles, $U_{z{\text{-}}mean}$, simulated by CFD for lumbar drain (left) and Neurapheresis therapy (right). **c** Steady-streaming velocity magnitude, $U_{ss}$, and **d** non-dimensional fraction of flow rate amplitude, $Q_{ss}$

Neurapheresis therapy was found to have a larger impact on $U_{z{\text{-}}mean}$ and $U_{ss} (z)$ in comparison to lumbar drain. Overall, Neurapheresis therapy resulted in greater steady-streaming velocity magnitude within the region between the return and aspiration ports (Fig. 4). The sagittal $U_{z{\text{-}}mean}$ velocity profile indicated a large region of caudally directed steady-streaming on the posterior side of the middle thoracic SAS during Neurapheresis therapy. $U_{z{\text{-}}mean}$ within this region decreased with lumbar drain. However, steady-streaming remained similar between the two therapies on the anterior side of the cervical SAS. The coronal $U_{z{\text{-}}mean}$ velocity profile was of a smaller magnitude and similar in both Neurapheresis therapy and lumbar drain (Fig. 4a). $U_{z{\text{-}}mean}$ indicated a cranially directed steady-streaming in the left frontal cranial SAS and caudally directed steady-streaming elsewhere. Steady-streaming in the cranial SAS was ~ 50× smaller than in the spinal SAS for both Neurapheresis therapy and lumbar drain (Fig. 4b). Note, to better visualize results along the entire spine, Fig. 4b is contracted at ½ scale in the z-direction (maximum spine curvature with respect to the z-axis is < 15 degrees). The average value of $U_{ss}$ between the aspiration and return ports was 60% greater with Neurapheresis therapy (0.37 mm/s versus 0.23 mm/s for lumbar drain) (Fig. 4c). $Q_{ss}$ showed a nearly identical trend as $U_{ss}$. The average value for $Q_{ss}$ between the aspiration and return ports was 0.040 and 0.025 for Neurapheresis therapy and lumbar drain, respectively (Fig. 4d).

The square of the Womersley number and oscillatory Peclet numbers were calculated to estimate the potential enhancement of dispersion by shear. For both tracer and hemoglobin $\alpha^{2}$ is 7.84 in the cortical SAS and 67.55 in the spinal SAS. The Womersley number is in the unsteady flow regime in the spinal SAS, but only marginally in the cortical SAS.

$\beta^{2}$ for tracer and hemoglobin is 1.64 E + 04 6.84 E+04 in the cortical SAS and 1.41 E+05 and 5.89 E+05 in spinal SAS. The large Peclet numbers indicate that dispersion is unsteady, thus secondary mixing across the cross section can increase axial dispersion.

$R_{max}$ for tracer and hemoglobin is 6.21 E+06 and 2.59 E+07 in the cortical SAS and 7.2 E+05 and 3.0 E+06 in the spinal SAS. Since $R_{max}$ is large compared to unity, the effective diffusivity is independent of molecular diffusivity. Therefore ${\mathbf{D}}$ is 0.0026 m^2^/s in the cortical SAS and 3.0677 E−04 m^2^/s in the spinal SAS. $Sh$ number for the tracer and hemoglobin was calculated to be 7.5 E−06 at the cortical SAS and 9.6 E−03 at the spinal SAS, respectively.

### Comparison of tracer concentration

**Fig. 5:**
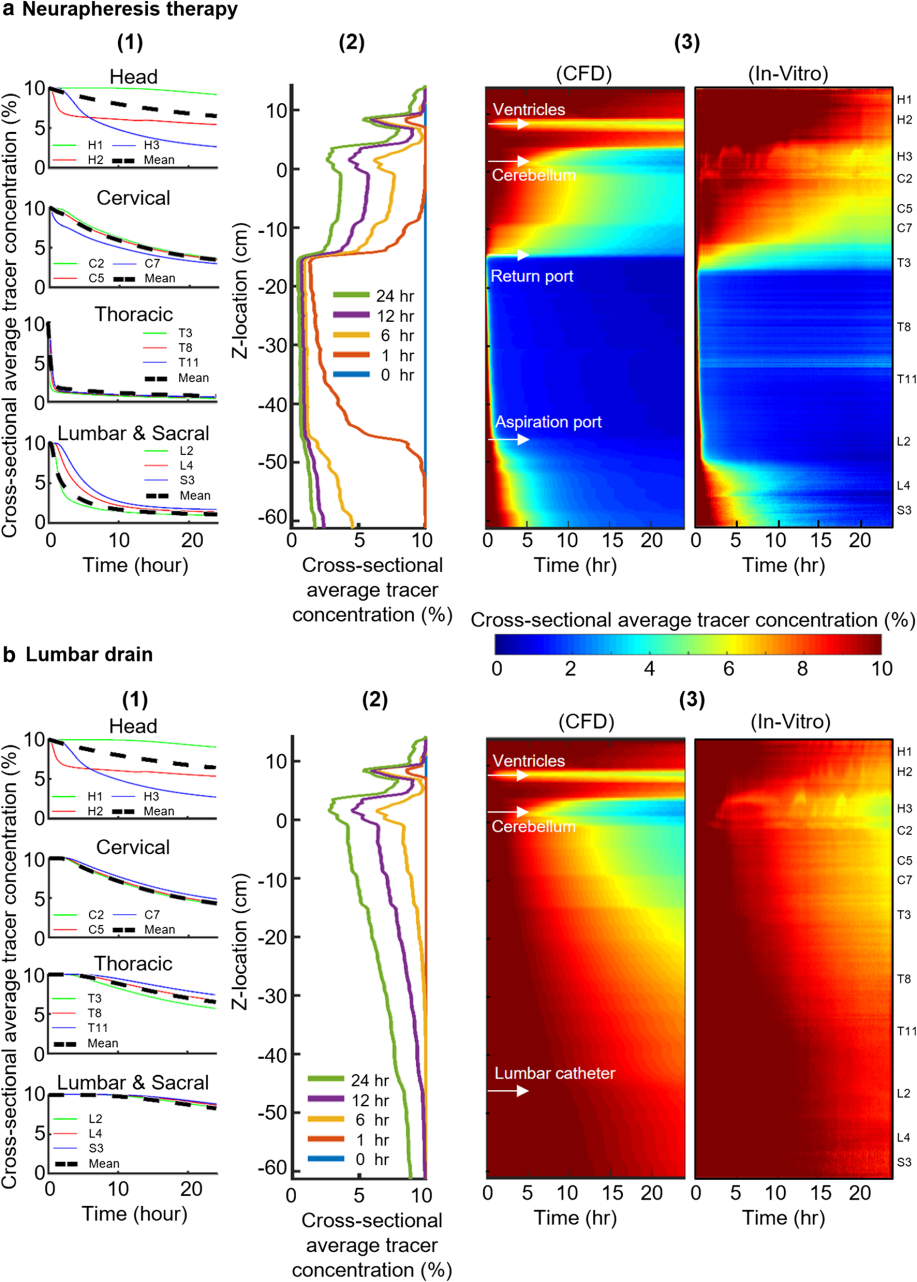
Cross-sectional average tracer concentration over 24-h. **a** Neurapheresis therapy and **b** lumbar drain. (a1) Cross-sectional average tracer concentration plotted with respect to time at specific axial locations under Neurapheresis therapy. (a2) Cross-sectional average tracer concentration along the neuroaxis for different time points, t = 0, 1, 6, 12, and 24 h under Neurapheresis therapy. (a3) spatial temporal plot for cross-sectional average tracer concentration for CFD and in vitro under Neurapheresis therapy along the model for 24-h. (b1) Cross-sectional average tracer concentration plotted with respect to time at specific axial locations under lumbar drain. (b2) Average tracer concentration along the neuroaxis for different time points, t = 0, 1, 6, 12, and 24 h under lumbar drain. (b3) spatial temporal plot for cross-sectional average tracer concentration for CFD and in vitro under lumbar drain along the model for 24-h

Baseline tracer concentration was set to 10% throughout the model. After 24-h, tracer concentration was reduced to 4.9% under Neurapheresis therapy compared to 6.5% under lumbar drain. Tracer clearance in the thoracic region occurred more rapidly after 1 h under Neurapheresis therapy compared to lumbar drain (Fig. 5a1, b1, Thoracic). There was little difference in the intracranial cross-sectional average tracer concentration for Neurapheresis therapy versus lumbar drain (6.6% in both cases) (Fig. 5a1, b1, Head). Cross-sectional average tracer concentration decreased to ~ 1.5% in the spinal SAS after 1 h (Fig. 5a2) compared to 6.5% with lumbar drain after 24-h (Fig. 5b2). Spatial–temporal distribution of tracer clearance under Neurapheresis therapy showed that maximum clearance occurred caudal to the return port (z = − 15 cm).

The highest deviations between the in vitro and CFD occurred in the cranial region (Fig. 5a3). The minimum tracer concentration in the cranial region occurred near the ventricles where the CSF production channels are located (~ z = 10 cm). Comparison of spatial–temporal tracer clearance trends with lumbar drain showed nearly identical results for both CFD and in vitro while the clearance rate decreased gradually in caudal direction (Fig. 5b3).

**Fig. 6:**
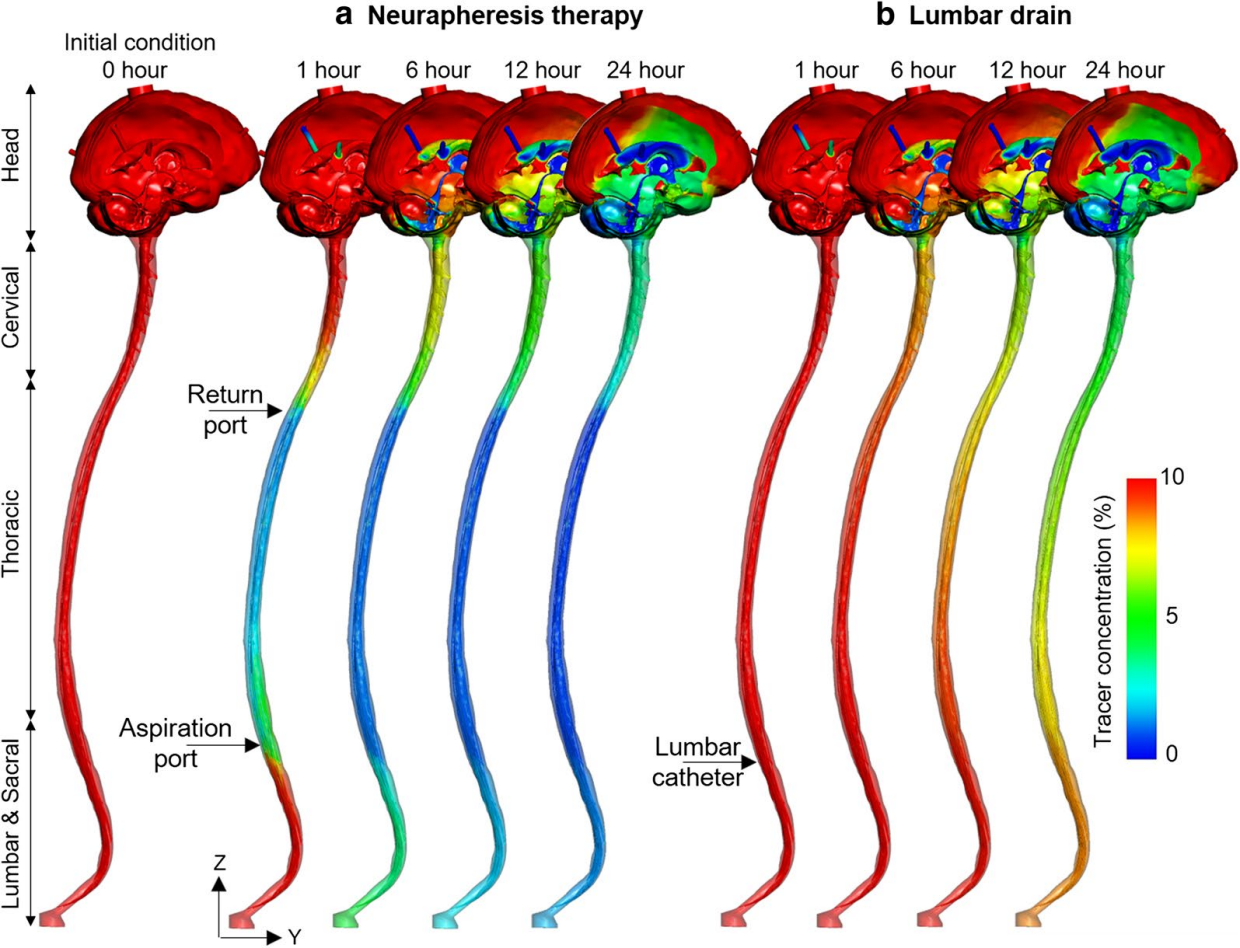
CFD results for 2D tracer concentration profiles versus time under Neurapheresis therapy and lumbar drain. **a** Visualization of tracer concentration at 0, 1, 6, 12, and 24 h under Neurapheresis therapy, and **b** visualization of tracer concentration at 1, 6, 12, and 24 h under lumbar drain

2D tracer concentration profiles were relatively uniform around the spinal cord circumference (X and Y directions) under both Neurapheresis therapy (Fig. 6a) and lumbar drain (Fig. 6b). In contrast, tracer concentration was non-uniform around the brain with local tracer concentration reduction near the cerebellum due to CSF production from the ventricles via the foramen Luschka and Magendie.

### Quantitative comparison of in vitro and numerical simulations

Overall, the distributions and clearance rates of tracer concentration in Neurapheresis therapy and lumbar drain match the bench-top patterns. Numerical simulations predicted slightly faster clearance rates under Neurapheresis therapy and lumbar drain than in vitro (Fig. 5).

**Fig. 7:**
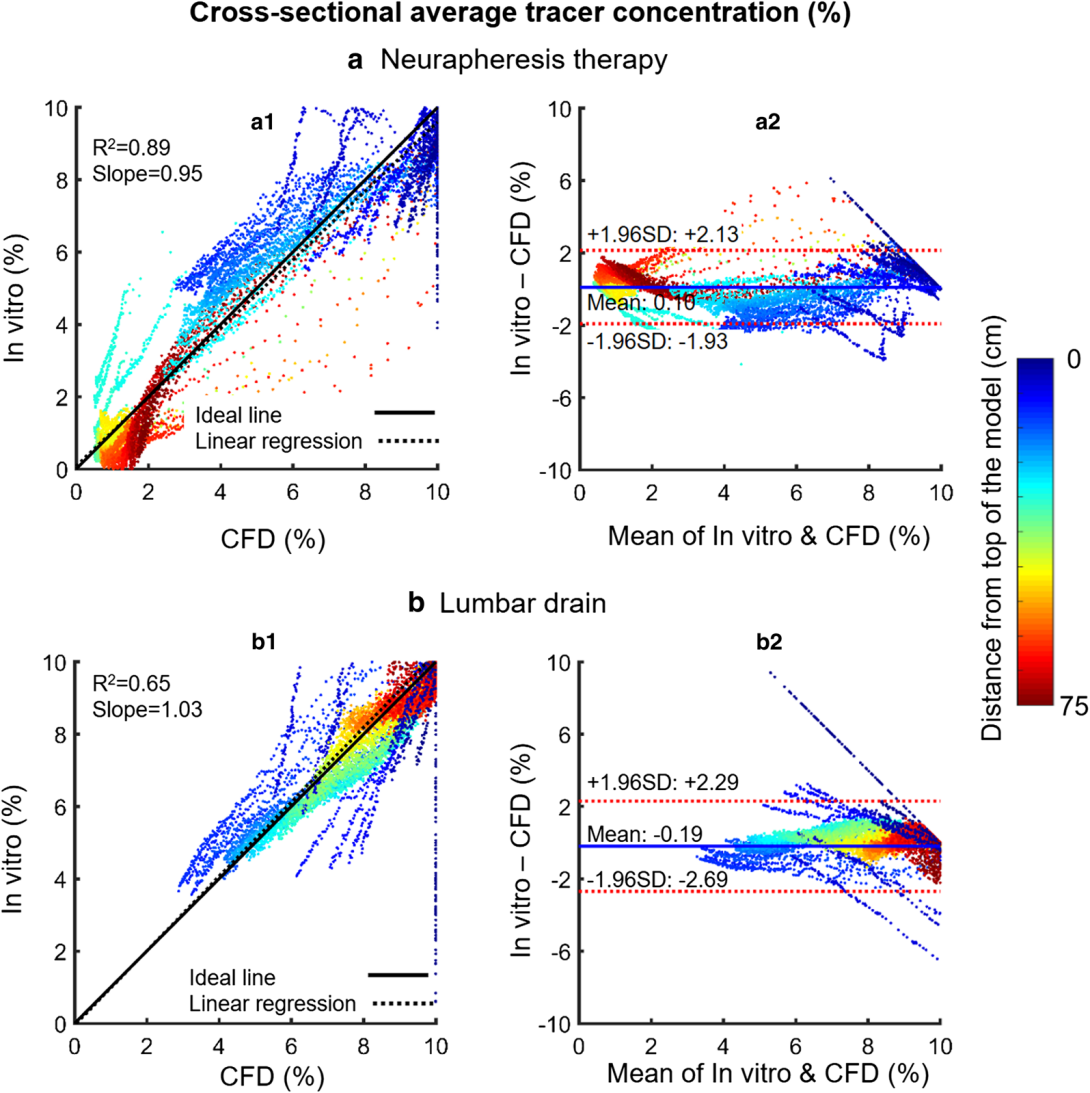
Correlation and Bland–Altman plots for agreement of in vitro and numerical simulation results for spatial–temporal cross-sectional average tracer concentration over 24-h. **a** Neurapheresis therapy and **b** lumbar drain. The linear regression is shown in black dashed line (left subplots, a1 and b1) and the limit of agreement (95% confidence intervals) lines are shown in dashed lines (right subplots, a2 and b2)

Differences between spatial–temporal cross-sectional average tracer concentration over 24-h obtained from in vitro and CFD were quantified using Bland–Altman plots (Fig. 7). A relatively strong linear correlation was observed between the numerical and in vitro results for Neurapheresis therapy (Fig. 7a1, $R^{2}$ = 0.89, slope = 1.01). Linear correlation for the lumbar drain case was moderate (Fig. 7b1, $R^{2}$ = 0.65, slope = 1.2). The second set of Bland–Altman plots (Fig. 7a2, b2) showed that a greater discrepancy between in vitro and CFD results tended to occur for z-positions closer to the cranium. The 95% confidence intervals for Neurapheresis therapy and lumbar drain were + 2.13 to − 1.93% and + 2.29 to − 2.69%, respectively (Fig. 7a2, b2).

## Discussion

The CFD and in vitro model provided a systematic comparison of lumbar drain to Neurapheresis therapy after SAH. These models allow detailed comparison of results without the confounding impact of many variables that would be difficult to control for in vivo animal models or SAH patients. Computer simulations provided the theoretical basis to interpret bench top in vitro results by better elucidating the complex tracer clearance patterns in pulsatile CSF after SAH.

### In vitro verification of numerical results

Studies show that wide variability exists in CFD modeling techniques and the choice of numerical solvers and settings are complex and can yield disparate results for biofluids simulations [53]. Thus, in vitro models play a critical role to help verify numerical results. Unfortunately, at present there is no known method to map exact spatial–temporal blood concentration within the CSF over time for SAH patients. Thus, a true model validation against in vivo measurements is not possible. The model results are presented as a prediction for how blood can potentially be removed from the CSF.

Comparison of spatial–temporal cross-sectional average tracer concentration profiles revealed similar clearance trends for both CFD and in vitro under Neurapheresis therapy and lumbar drain conditions (Fig. 5). A strong linear correlation was found between CFD and in vitro results under Neurapheresis therapy (R^2^ = 0.89, Fig. 7a1), and a moderate linear correlation for lumbar drain (R^2^ = 0.65, Fig. 7b1). Lumbar drain correlation was lower likely due to the lower degree of tracer changes that were present in that experiment. It was noted that linear correlation of results was stronger within the central region of the models. We expect that results had improved agreement within the central region of the model because our optical imaging field of view was most accurately aligned to CFD results within that region of the model. Near the model ends, the camera viewing angle was not orthogonal to the model domain and therefore did not provide information in the exact axial z-orientation as CFD results. Even with these in vitro imaging limitations, CFD results showed 95% of the CFD tracer concentration results were within ~ 2% of the in vitro findings with a mean difference of ~ 0.1% in both cases (Fig. 7a2, b2). In combination, these results help verify the numerical modeling approach using a frozen flow field that excluded mass diffusion. While these results agree, they cannot be assumed to correctly represent in vivo as many model assumptions were made that may not exactly represent in vivo CSF mass transport (see “Limitations”). These model predictions should be tested against in vivo measurements in animals and/or humans.

Our model results are difficult to directly compare with previous research as no study has been conducted previously with an anatomically realistic model and with Neurapheresis therapy applied with an exact catheter geometry. However, Tangen et al. [37] used an anatomically idealized bench-top CSF model and corresponding CFD analysis to study CSF blood clearance following SAH under various body orientations and lumbar drain rates with an intraventricular catheter inserted for 3 h. They found the fastest blood clearance was achieved in the vertical body position and that an increase in lumbar drainage flow rate accelerated blood clearance. Their results, using a lumbar drain and intraventricular catheter, showed that after 60 min of filtration, contamination concentration was 3.5% at the T6 vertebral level. After 60 min, tracer clearance was 1.5% at T6 in our numerical model using Neurapheresis therapy (Fig. 5a1). This difference is likely due to the 2× higher filtration rate applied in our study 2.0 mL/min versus 1.0 mL/min by Tangen et al. Also, for the lumbar drain case with 0.2 mL/min drainage rate, 12% clearance was observed in Tangen et al. [37] versus 10% clearance in our simulation. Since drainage rate for lumbar drain is equal on both studies, the clearance rates are similar.

### Comparison of Neurapheresis therapy and lumbar drain

After 24 h, results from Neurapheresis therapy showed that 4.9% of tracer remained in the model (Fig. 6a) while 6.5% tracer concentration remained after lumbar drainage (Fig. 6b). Cranial tracer clearance was nearly identical in both the lumbar drain and Neurapheresis therapy (Fig. 5a1, b1). The mechanistic reason for increased tracer clearance under Neurapheresis therapy is that it applies a CSF flow loop that returns filtered CSF back to the upper thoracic spine. The CSF flow loop increases steady-streaming velocities within the flow loop region (Fig. 4), which allows more rapid removal of the tracer. While the clinical impact of greater blood clearance on SAH outcomes has not been proven, researchers have shown the potential that more quickly reducing the levels of blood and inflammatory cytokines in the CSF post SAH could improve outcomes [54, 55]

The Neurapheresis therapy flow rate applied in our study was 2.0 mL/min with a 1.8 mL/min return flow rate. A flow rate of 2.0 mL/min is not possible to apply using a lumbar drain because it would remove CSF more rapidly than it is being produced at the choroid plexus (~ 500 mL/day) [56]. To help compare Neurapheresis and lumbar drain tracer clearance efficiency, we compared tracer clearance under a lumbar drain and Neurapheresis waste rate both set to 0.2 mL/min (288 mL in 24 h). To the best of our knowledge, this flow rate represents an upper bound for what is possible to withdraw under lumbar drain. In clinical practice, the drainage rate settings for lumbar drains may be lower.

### Importance of frozen field approach in the numerical model

Transient simulations of oscillating fluids are computationally intensive, in particular when conducted over long time periods with small time-step size. For example, in the present case representing CSF oscillations, computation of a single CSF flow cycle requires ~ 3.6 h using 38 processors (Intel(R) Xeon(R) Gold 6148 CPU @ 2.40 GHz) and 126 (GB) Memory. Simultaneously solving the passive transport equation requires additional time. Neurapheresis therapy is conducted over a period of more than 24-h. As such, we applied a two-part CFD method that neglected diffusion to obtain a computationally tractable solution over the 24-h timeframe. First, a transient Navier–Stokes solution of 11 flow cycles was performed to obtain the steady-streaming velocity field. Steady-streaming is postulated to be responsible for the time-average bulk movement of CSF in the SAS that results from nonlinear cumulative effects of convective acceleration [57]. Steady streaming is important in this context because it has been shown to be the primary mode of mass transport within the oscillatory CSF flow field [58]. Second, the velocity field was applied as a “frozen flow field” as described by Kuttler et al. [40]. The frozen field approach is valid for periodic flow when advection is the main mode of mass transport.

The $Sh$ numbers computed in our study, by using the effective diffusivity of the tracer, were 7.56 E−06 and 9.6 E−03 for the cortical and spinal SAS, respectively. It should be noted that the low Sherwood number based on $R_{max}$ does not necessarily convey that shear-augmented diffusion is important, in particular for the present case in which substantial mixing can be produced by the complex spinal cord nerve root geometry. Further study is needed to compare the effect of diffusion to steady-streaming based advection.

In this study, we did not include the potential impact of microscopic anatomy within the domain such as arachnoid trabeculae or blood vessels, nor hydrodynamic affects on finite-size particles (red blood cells, in particular). Other numerical studies have investigated the potential impact of microscopic structures [41] within the CSF and found they can have varying degrees of impact on solute transport [59–61] and pressure gradients [62]. Thus, our numerical and in vitro predictions should be confirmed with in vivo experiments. Albeit, these experiments may not be possible at present as we do not have a non-invasive in vivo imaging modality that can quantify blood concentration throughout the CSF system over 24-h.

### Limitations

In this study, blood dispersion was modeled by fluorescein tracer mixed in a single continuum CSF phase at room temperature. Physiologically, blood cells and debris create a suspension when mixed into CSF. The biochemistry of blood coagulation within the CSF was not reproduced. Additionally, once exposed to the SAS environment, blood cells can rupture releasing oxyhemoglobin which is further enzymatically converted to bilirubin [63, 64]. While the electrolytes and enzymatic interactions between blood components and CSF have an impact, our fluid mechanical study did not take into account pharmacokinetics of blood proteins, blood cell lysis, and blood cell component metabolism. Accounting for red blood cell byproducts and reaction kinetics could provide a more realistic scenario for testing biochemical effects of SAH. However, the effective diffusivity is independent of molecular diffusivity since R_max_ is large compared to unity. Therefore, the chosen tracer provides good similitude for blood. The in vitro experiments were performed at 19 °C. We did not create a thermostatic environment due to size limitations, because the density ratio between CSF and fluorescein tracer is not different whether the experiment is conducted at 19 °C or at body temperature of 37 °C.

In the comparison between simulation and experiments, the highest deviations occurred in the cranial SAS. It is likely these differences were larger in the cranial SAS due to the 2D imaging technique that used a picture obtained for a single angle relative to the model, whereas, the CFD concentrations were precisely averaged across each 3 mm thick slice, including fluid located within the ventricles of the brain. Future work could potentially improve agreement of in vitro and numerical results by utilizing tomographic projection imaging [65] of the in vitro model or quantitative contrast enhanced MRI techniques [66].

The numerical simulations in this study were based on MRI measurements for a single subject-specific CSF system geometry and CSF flow waveform. These parameters should be investigated in a larger cohort to determine the potential impact of age, sex, and disease states on CSF solute transport. However, the consistency of CSF dynamics across humans in the healthy state and with ALS has been studied by our group and we found relatively small differences across subjects [67]. Therefore, we expect our results would hold true for other human cases with slightly different CSF space geometry. Also, for future research, we may need to investigate the effect of filtration for a longer periods of 48, 72 or 120 h for different neurological conditions [68].

Our modeling approach did not include flow oscillations within the ventricles [69, 70] or a respiratory component of CSF pulsations [71–73] because the MRI scanning time did not allow measurement of these parameters in addition to the other parameters used to formulate the model. Additionally, the presented model used a rigid material in which boundary motion of the dura was not prescribed [25]. This model also did not account for permeability of the CNS tissue or dura matter [74]. We chose a rigid model without permeability to allow verification of numerical results in a precisely known domain. Future studies should investigate the relevance of tissue permeability and motion.

Our model only had one single site of CSF production in the lateral ventricles because the focus of our study was on CSF solute transport within the subarachnoid space, external to the ventricles, we simplified CSF production to occur at a single site within the lateral ventricle. CSF production was assumed to flow out into the cisterna magna where mixing occurs with CSF in the subarachnoid space. Also, the in vitro system did not allow imaging of tracer concentration within the ventricles, and therefore we were not able to compare in vitro to computational results within the ventricles. Future studies should investigate the impact of CSF production location by adding the choroid plexus in the third and fourth ventricles.

No attempt was made in this study to optimize catheter design or positioning for Neurapheresis therapy. The effect of Neurapheresis therapy on CSF steady-streaming velocities in the spinal SAS were investigated in our previous study [21]. The present study extended the previous model by including a complete CSF system, integration of a two-phase model, and developing a method for in vitro verification of results.

## Conclusions

A subject-specific CFD model of the CSF system was formulated and applied to compare the impact of Neurapheresis therapy on tracer removal from CSF compared to lumbar drain over a 24-h period. Results were verified with an in vitro model built identical to the CFD model. The numerical modeling approach using a frozen flow field to represent solute transport resulted in similar solute transport dynamics as that seen in vitro. Using the verified computational model with in vitro system, the results predict that Neurapheresis therapy significantly increases tracer clearance compared to a lumbar drain. The overall tracer concentration after a 24-h period for Neurapheresis therapy was 4.9% compared to 6.5% with lumbar drain. This effect was maximized within the region between the return and the aspiration ports in Neurapheresis therapy.

## Data Availability

The data that support the findings of this study are available from the corresponding author, [BAM], upon reasonable request.

## Abbreviations

Ρ: Density (kg/m^3^)
Μ: Dynamic viscosity (Pa s)
Ν: Kinematic viscosity (m^2^/s)
Α: Volume fraction
A: Area
D: Diffusion coefficient
H: Convective mass transport
I: Signal intensity
L: Characteristic length
Q: Flow rate
U: Velocity
V: Cell volume
D_H_: Hydraulic diameter
Re: Reynolds number
Sc: Schmidt number
Sh: Sherwood number
CFD: Computational fluid dynamics
CNS: Central nervous system
CSF: Cerebrospinal fluid
MRI: Magnetic resonance imaging
SAH: Subarachnoid hemorrhage
SAS: Subarachnoid spaces
